# Optimal selection for *BRCA1* and *BRCA2* mutation testing using a combination of ‘easy to apply’ probability models

**DOI:** 10.1038/sj.bjc.6603306

**Published:** 2006-08-15

**Authors:** D Bodmer, M J L Ligtenberg, A H van der Hout, S Gloudemans, K Ansink, J C Oosterwijk, N Hoogerbrugge

**Affiliations:** 1 Department of Human Genetics, Radboud University Medical Centre Nijmegen, PO Box 9101, 6500 HB Nijmegen, The Netherlands; 2 Department of Pathology, Radboud University Medical Centre Nijmegen, PO Box 9101, 6500 HB Nijmegen, The Netherlands; 3 Department of Clinical Genetics, University Medical Centre Groningen, Groningen University, PO Box 30.001, 9700 RB Groningen, The Netherlands

**Keywords:** breast cancer, ovarian cancer, *BRCA1*, *BRCA2*, mutation, probability model

## Abstract

To establish an efficient, reliable and easy to apply risk assessment tool to select families with breast and/or ovarian cancer patients for *BRCA* mutation testing, using available probability models. In a retrospective study of 263 families with breast and/or ovarian cancer patients, the utility of the Frank (Myriad), Gilpin (family history assessment tool) and Evans (Manchester) model was analysed, to select 49 *BRCA* mutation-positive families. For various cutoff levels and combinations, the sensitivity and specificity were calculated and compared. The best combinations were subsequently validated in additional sets of families. Comparable sensitivity and specificity were obtained with the Gilpin and Evans models. They appeared to be complementary to the Frank model. To obtain an optimal sensitivity, five ‘additional criteria’ were introduced that are specific for the selection of small or uninformative families. The optimal selection is made by the combination ‘Frank ⩾16% or Evans2 ⩾12 or one of five additional criteria’. The efficiency of the selection of families for mutation testing of *BRCA1* and *BRCA2* can be optimised by using a combination of available easy to apply risk assessment models.

Identification of families at high risk of hereditary breast and/or ovarian cancer contributes to the prevention and early detection of breast and ovarian malignancies. Therefore, genetic testing is offered to women with an increased risk of hereditary breast or ovarian cancer based on familial clustering of breast and/or ovarian cancer, particularly in case of early onset or if breast cancer occurs in a male. Selection criteria to test for *BRCA* mutations vary. Because *BRCA* testing is laborious and expensive, as well as associated with medical, psychological and social consequences for the patient, careful patient selection is required before testing. To obtain optimal ascertainment, many risk assessment models and prior probability models have been developed and evaluated (de la Hoya *et al*, 2003; Domchek *et al*, 2003). Four such models, the Claus, Gilpin, Frank and Evans model (Claus *et al*, 1998; Gilpin *et al*, 2000; Frank *et al*, 2002; Evans *et al*, 2004) are empirically derived scoring systems, easy to apply in daily practice with the use of a pencil and a paper and easy to understand for both counsellor and patient.

With the Claus tables the probability of developing breast cancer can be determined, but not the likelihood of detecting a *BRCA* mutation (as in prior probability models). These tables are based on series of unselected women with breast cancer. As this model does not account for breast cancer in more than two family members, the presence of ovarian cancer, male breast cancer or bilateral breast cancer, it underestimates the cancer risk in many families and cannot be used solely as a prediction model in the clinic.

The Frank model, developed by Myriad Genetics, is an empirical model correlating the prevalence of *BRCA* mutations with personal and family history of breast and ovarian cancer and is based on thousands of women tested for *BRCA1* and *BRCA2* in a diagnostic setting. The outline of this scoring system is shown in Table 1.

Both previous models do not account for the presence of bilateral breast cancer, although this is a predictor for the presence of *BRCA* mutations (Ligtenberg *et al*, 1999). Gilpin *et al* (2000) defines a family history assessment tool (FHAT) that does include bilaterality and ovarian cancer as well as other suggested variables that are prevalent in *BRCA* families, like prostate cancer and colon cancer diagnosed before age 50 (Bermejo and Hemminki, 2004; Table 1).

Like Gilpin *et al* (2000), Evans *et al* (2005) developed a simple and quick scoring system (Manchester scoring system). This model was developed using empirical data from families tested for *BRCA1* or *BRCA2* mutations. The scoring mainly depends on the type of cancer and age at diagnosis and is different for the prediction of *BRCA1* and *BRCA2* mutation-positive families (Evans1 and Evans2 scores; Table 1). The 10 point cutoff level identifies a >10% likelihood to find in particular *BRCA2*, but also *BRCA1* mutations.

Although the number of *BRCA* analyses increased dramatically over the last 10 years, the number of mutation-positive results did not follow this increase. To reduce the number of negative test results, we correlated the outcome of the aforementioned simple, quick and easy to apply prediction models on families with breast and/or ovarian cancer previously screened for *BRCA* mutations with the mutation status. The sensitivity of the selection criteria was subsequently improved by changing cutoff levels, combining models, and by adding five so-called ‘additional selection criteria’. This new combination of selection criteria for *BRCA* testing was validated on two other sets of *BRCA* mutation tested families from two different clinics.

## PATIENTS AND METHODS

### Study population

The cohort studied consists of 263 families with breast and/or ovarian cancer patients that were tested for *BRCA* mutations, after thorough genetic counselling, between 1999 and 2001, at the Radboud University Medical Centre Nijmegen. Information about the family history was reported to a clinical geneticist by the index patient, that is, the affected family member who was considered to have the highest prior probability of carrying a *BRCA* mutation and who was the first to be tested in the family. The pedigrees were anonymised before analysis and cancer diagnoses were verified wherever possible. In the period 1999–2001, the selection of families for DNA testing was mainly based on expert opinion of clinical geneticists, using the Claus tables (selection when breast cancer risk was three times the population risk: ⩾30%). In 2001 the Gilpin and Frank models were added to the Claus tables and genetic testing was offered if one of these scores was ⩾10. In this study, we used the Myriad mutation prevalence tables of 1 August 2002 (Frank model; http://www.myriadtests.com/provider/mutprev.htm).

Validation of the thus defined combination of selection criteria and cutoff levels was performed in two additional family sets. One consisted of 197 breast/ovarian cancer families counselled and tested in 2002 at the Radboud University Medical Centre Nijmegen, of which 34 families were tested *BRCA* mutation-positive. The other set consisted of 101 *BRCA* mutation-positive families counselled and tested at the University Medical Centre Groningen.

### Mutation detection in *BRCA1* and *BRCA2*

The mutations were identified by analysis of the entire coding sequences and intron/exon boundaries of the genes using a combination of techniques: protein truncation test of *BRCA1* and *BRCA2* exon 11; denaturing gradient gel electrophoresis of the other coding exons including the 5′ and 3′ regions of the exons 11 (Hout van der *et al*, 1999); and multiplex ligation-dependent probe amplification to detect exon deletions or duplications (Schouten *et al*, 2002; Hogervorst *et al*, 2003). All positive tests were confirmed by direct sequencing. Unclassified variants were not included in this study.

### Scoring systems

The Claus, Frank, Gilpin and Evans scores were determined following description in the published papers. The Evans scores were calculated up till third degree family members.

### Data analysis

The significance of the difference between the median prediction scores determined with the different models and correlated with the mutation status of these families was calculated with a Mann–Whitney *U*-test. We evaluated the different prediction scoring systems and different cutoff levels by calculating the sensitivity, specificity and predictive values. Sensitivity of the selection criteria is the proportion of *BRCA* mutation carriers who fulfil the selection criteria (higher than or equal to the cutoff level). Specificity is the proportion of noncarriers who do not fulfil the selection criteria (below the cutoff level). The positive predictive value is the proportion of individuals who fulfil the selection criteria and who carry a *BRCA* mutation. The negative predictive value is the proportion of individuals who do not fulfil the selection criteria and who do not carry a *BRCA* mutation. We calculated the probability of a *BRCA* mutation for each woman. Subsequently, these probabilities were used to construct a receiver operating characteristic (ROC) curve in order to visualise the predictive power of the variables. The area under the ROC curve (C statistic) quantifies this predictive power.

## RESULTS

To determine the *BRCA* mutation prediction capability of the Claus, Frank, Gilpin, Evans1 and Evans2 models, the prediction scores were determined for 263 families with breast and/or ovarian cancer patients and compared to the mutation status of these families. For both the *BRCA-*positive and -negative families, the median prediction scores for the five models were calculated. For the Claus model, no significant difference in this score was observed between *BRCA*-positive and -negative families (*P*=0.168). The Frank, Gilpin, Evans1 and Evans2 model showed a significant difference in the prediction scores (*P*<0.0001) and were studied in more detail.

**Figure 1:**
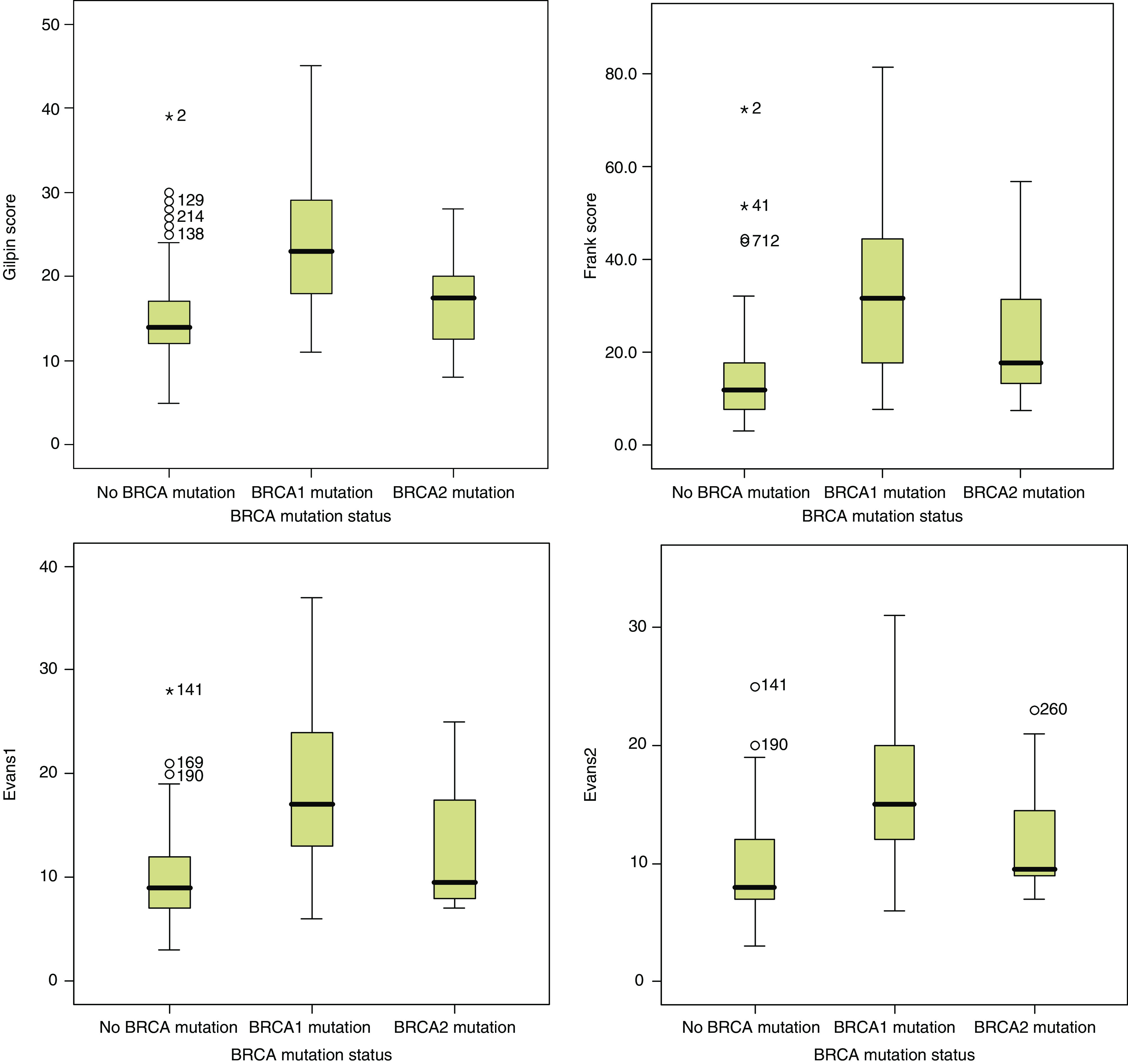
Boxplot graphs from the Gilpin, Frank, Evans1 and Evans2 scores for the different families and their *BRCA* mutation status (*BRCA*1 or *BRCA*2 mutation negative; *BRCA*1 mutation-positive; *BRCA*2 mutation-positive). The line in the box indicates the median value of the data. The box itself contains the middle 50% of the data. The ends of the vertical lines indicate the minimum and maximum data values. The points outside the ends are outliers.

The prediction scores were also determined for *BRCA1* and *BRCA2* mutation-positive families separately (Figure 1). In all models, the prediction score for the *BRCA1* families was significantly better than for the *BRCA2* families (*P*-value varied from 0.008 for the Gilpin and Evans1 models to 0.047 for the Frank model). For the Gilpin model, the difference in the prediction scores between *BRCA2* families and noncarriers was not significant (*P*=0.113). The Evans2 gave the best difference between *BRCA2* families and noncarriers (*P*=0.012). All models did distinguish significantly the *BRCA1* families from the noncarriers (*P*<0.0001).

In order to visualise the predictive power of each model, ROC curves were generated for all models and the areas under the curves (C statistic) were determined. The Evans1 model outperformed all other models for the prediction of *BRCA1* mutation-positive families. The Evans2 model performed best for the prediction of *BRCA2* mutation-positive families. For all models we assessed the sensitivity for *BRCA1* mutation-positive families and *BRCA2* mutation-positive families for the advised cutoff levels (⩾10 for all models). Thus, the Gilpin model scored best (sensitivity *BRCA1*: 100%; sensitivity *BRCA2:* 88%, two families missed). The Evans1 and Evans2 model scored disappointing for *BRCA2* families. Only 50% of all *BRCA2* families were selected. For *BRCA1* families, the Evans1 and Evans2 selection scored much better and only one family was missed. With the Frank model, four *BRCA1* mutation-positive families were missed (sensitivity of 88%) and four *BRCA2* mutation-positive families (sensitivity of 75%).

To improve the efficiency of selection for *BRCA* mutation testing, the sensitivity and specificity were calculated for various cutoff levels in all models (Table 2 and Supplementary data Table 1). As expected, the sensitivity decreased and the specificity increased when higher cutoff levels were used. However, no clear optimal combination of sensitivity and specificity could be reached. Scatter plots revealed that the models complement each other in several families (data not shown). Therefore, combinations of different cutoff levels of the two models were tested (Table 2 and Supplementary data Table 2).

Overall, the combination of two prediction scores had a higher sensitivity than each individual model although the specificity decreased. ‘Frank ⩾16 or Evans2 ⩾12’ showed the to be the best combination with respect to highest sensitivity and specificity (96 and 47%, respectively; Table 2). Only two mutation-positive families were missed. These two families were *BRCA2* mutation-positive families. In one of these families, a mother and daughter had developed breast cancer at age 56 and 26, respectively (Frank: 7.8%; Gilpin: 6; Evans1: 8; Evans2: 7). In the other family, two sisters had breast cancer at the ages 47 and 52 and one brother developed pancreatic cancer (Frank: 8.3%; Gilpin: 8; Evans2 (and 1): 5). Both families had very few female relatives. The combination of ‘Frank ⩾16 or Gilpin ⩾16’ had an almost equal sensitivity and specificity of 94 and 40%, respectively (Table 2).

To include such *BRCA* mutation-positive cancer families, five ‘additional criteria’ were introduced that are focussed on individual features of one affected person in a family at risk (Table 3). These criteria include all relatives from the index patient up till the third degree. Sensitivity, specificity and predictive values were calculated for all models and model combinations in combination with these ‘additional criteria’ (Table 2 and Supplementary data Table 3). To obtain an optimal sensitivity, we also varied the cutoff levels.

An optimal combination of sensitivity and specificity was obtained with the combination ‘Frank ⩾16 or Evans2 (or 1) ⩾12 or one of the additional criteria’. However, this combination missed the same *BRCA2* mutation-positive family (the two sisters with breast cancer at the ages 47 and 52 and one brother with pancreatic cancer) as none of the ‘additional criteria’ were fulfilled. To obtain 100% sensitivity, the cutoff levels should be lowered such, that there is hardly any specificity gained relative to the initial selection based on expert opinion of clinical geneticists.

The specificity and positive predictive value were best for the combination ‘Frank ⩾16 or Evans1 (or 2) ⩾12 or one of the additional criteria’. With this selection combination, 22% less families would have been tested for *BRCA* mutations than without these criteria. With the selection combination ‘Frank ⩾16 or Gilpin ⩾16 or one of the additional criteria’, this profit would have been 18%.

### Validation

The selection combinations, ‘Frank ⩾16 or Gilpin ⩾16 or one of the additional criteria’ and ‘Frank ⩾16 or Evans1 (or 2) ⩾12 or one of the additional criteria’, were validated in a cohort of 197 breast/ovarian cancer families tested in 2002. With the combination ‘Frank ⩾16 or Gilpin ⩾16 or one of the additional criteria’ only one *BRCA2* family was missed. The same *BRCA2* family was also missed with the combination ‘Frank ⩾16 or Evans1 ⩾12 or one of the additional criteria’. The combination ‘Frank ⩾16 or Evans2 ⩾12 or one of the additional criteria’ missed besides this *BRCA2* family also one *BRCA1* family. The specificity for these combinations varied between 18 and 21% and is less than that for the test group of 263 individuals. This difference may be due to a difference in selection stringency of both groups. The families that have been tested for *BRCA* mutations in 2002 scored higher median selection scores for all models than the families that have been tested between 1999 and 2001. The more stringent selection in 2002 thus explains the lower specificity in these analyses. Despite of that, about 15–17% of all tested families would not have been tested with these new selection combinations.

In the *BRCA2*-positive family that was missed with both the selection combinations ‘Frank ⩾16 or Gilpin ⩾16 or one of the additional criteria’ and ‘Frank ⩾16 or Evans1 ⩾12 or one of the additional criteria’, the index case had developed breast cancer at age 68. The mother and grandmother were both diagnosed with breast cancer at ages 37 and 40, respectively, but had died and could not be tested (Frank: 12%; Gilpin: 15; Evans1 (and 2): 8). If the mother would have been chosen as the index, the Frank and Gilpin scores would have been higher than 16 and thus this family would have been included in the selection for *BRCA* testing.

A second validation was performed for the combination ‘Frank ⩾16 or Gilpin ⩾16 or one of the additional criteria’ in 101 *BRCA*-positive families genetically tested at the University Medical Centre Groningen (70 families with a *BRCA1* mutation and 31 families with a *BRCA2* mutation). Only one *BRCA2*-positive family was missed. In this family the index had developed breast cancer at age 61. Two sisters had also developed breast cancer but the ages were not known and these variables could therefore not be added in the scores. In this family, the lack of information is the cause of not meeting the selection criteria. This family would also have been missed with ‘Frank ⩾16 or Evans2 (or 1) ⩾12 or one of the additional criteria’.

## DISCUSSION

Efficacy of *BRCA1* and *BRCA2* mutation testing can be improved by selection of families using both a combination of existing probability models as well as higher cutoff levels than advised in the previous publications. With a combination of easy to apply probability models: ‘Frank ⩾16 or Evans ⩾12 or one of the additional criteria’ a sensitivity of 98% was obtained with a substantial reduction in the number of selected families for *BRCA* testing as compared to the isolated use of one of these probability models at cutoff level 10.

In our daily practice, the number of *BRCA* mutation analysis is rapidly growing. However, the percentage of families with a detected *BRCA* mutation is decreasing. This urged us to improve the efficacy of the selection for mutation testing under the precondition that no mutation carrying family is allowed to be missed. In our clinical genetic setting, five existing probability models for the selection of *BRCA* mutation-positive families were analysed: Claus, Frank, Gilpin, Evans1 and Evans2. The latter four showed a good correlation with the mutation status and were studied in more detail. Most risk assessment and prior probability models are based on two or more affected family members and thus form a general limitation for the selection of families that lack family history information or families with only a few women. Addition of so-called ‘additional criteria’ to these models increased the sensitivity to almost 100%. These ‘additional criteria’ are applicable for one affected individual. We applied these ‘additional criteria’ for all affected members in a family up to the third degree of the index person. In case no family member fulfils one of the ‘additional criteria’, the selection is dependent on the Frank, Gilpin or Evans score.

The prevalence of *BRCA1* and *BRCA2* mutations among all women diagnosed with an invasive breast cancer or ductal carcinoma *in situ* is similar, and varies between 0.4–2.6% and 1.4–2.4%, respectively (Peto *et al*, 1999; Sanjose *et al*, 2003; Claus *et al*, 2005). However, both in our settings as well as in the literature, the families known to carry a *BRCA1* mutation outnumber those with a *BRCA2* mutation by far (Newman *et al*, 1998; Syrjakoski *et al*, 2000; Claus *et al*, 2005). This may mean that either *BRCA2* mutations are less common or that a considerable number of families actually carrying a *BRCA2* mutation are missed, most likely because they are not recognised by the current probability models. Consequently, a specific *BRCA2* prediction model is needed. *BRCA2* families differ less from *BRCA*-negative mutation families than *BRCA1* families do and may therefore need stricter or different selection criteria. Additionally, an explanation may be found in the fact that mutations in either of the two genes are related to different life time risks for nonbreast cancers such as ovarian cancer and prostate cancer (Antoniou *et al*, 2003; Van Asperen *et al*, 2005). Evans *et al* (2004) developed the so-called Evans2 model for the selection of *BRCA2* mutation-positive families. In our study, this model was analysed for different combinations and cutoff levels. Although ‘Gilpin ⩾16’ scored most *BRCA2* mutation-positive families compared to the other models, the Evans2 model showed the best accuracy for this type of families (area under the ROC curve). However, in our study group, 50% of the *BRCA2* families were missed in case the previously published cutoff level of 10 was used.

The sensitivity for the selection of *BRCA2* mutation-positive families of the Evans2 model was strikingly lower in our study as compared to the study by Evans *et al*. This difference may be explained by difference in selection of the study population or by a difference in carrier frequency between the British and the Dutch population.

One family from the validation group with a *BRCA2* mutation was missed by the Frank model, because the index patient was older than 50 years. The same family was also missed by the Gilpin model as the family relation with affected relatives was too distant (mother and grandmother from the affected index developed breast cancer). When in this case the mother was chosen to be index, the family would score ⩾16 for both the Gilpin and Frank model and thus would fulfil our selection criteria. This underlines that application of the selection models for *BRCA* mutation testing preferably should start in a family member with either breast cancer at the youngest age or with bilateral breast cancer or with ovarian cancer. *BRCA* mutations can be missed when the person with the highest probability to carry a mutation is not used as index in applying the selection model.

During recent years many studies describing new risk assessment models, prior probability models or comparisons of the performance of some existing models have been published (De la Hoya *et al*, 2003; Domchek *et al*, 2003; Marroni *et al*, 2004). A well-known model is BRCAPRO. This program was validated retrospectively on families with and without a *BRCA1* or *BRCA2* mutation (Parmigiani *et al*, 1998; Berry *et al*, 2002). To use this model a computer is needed. We did not include it in this study aimed to determine optimal prediction using a simple, quick and easy to apply probability model. In the present study we succeeded to optimise existing easy applicable models by combining them and adjusting the cutoff levels to a more specific and sensitive selection.

## Figures and Tables

**Table 1:** Outlines of the Frank, Gilpin and Evans scoring systems for the *BRCA* mutation prediction

**Prediction model**	**Outlines**
Frank	• Prevalence of mutations in *BRCA1* and *BRCA2* correlated with personal and family history of cancer in thousands of women tested for *BRCA1* and *BRCA2* in a diagnostic setting.
	• Family history includes at least one first- or second-degree relative and excludes proband
	*Characteristics scored for*
	• Age of onset breast cancer (< or ⩾50 years)
	• Ovarian cancer at any age
	• Combined breast and ovarian cancer within one patient
	
Gilpin	•Weighting system in which points are given to certain characteristics of cancers in affected individuals. The points is predictive for the mutation status
	•Family history includes all first, second and third degree family members
	*Characteristics scored for*
	• Age of onset breast cancer (20–29; 30–39; 40–49 years)
	• Age of onset ovarian cancer (<40; 40–60; >60 years)
	• Age of onset prostate cancer (< or ⩾50 years)
	• Age of onset colon cancer (< or ⩾50 years)
	• Bilateral/multifocal breast tumours
	• Combined breast and ovarian cancer within one patient
	• Male breast cancer
	
Evans (*BRCA1* and *BRCA2* scored separately)	• Separate scoring system for *BRCA1* and *BRCA2* mutation prediction
	• Weighting system in which points are given to certain characteristics of cancers in affected individuals. The points is predictive for the mutation status
	• Family history includes all first, second and third degree family members
	• Each breast cancer if bilateral/multifocal is counted separately
	*Characteristics scored for*
	• Age of onset breast cancer (<30; 30–39; 40–49; 50–59; >59 years)
	• Age of onset ovarian cancer (< or ⩾60 years)
	• Age of onset prostate cancer (< or ⩾60 years) (for *BRCA2* only)
	• Pancreatic cancer at any age (for *BRCA2* only)
	• Male breast cancer (for *BRCA2* only)

**Table 2:**
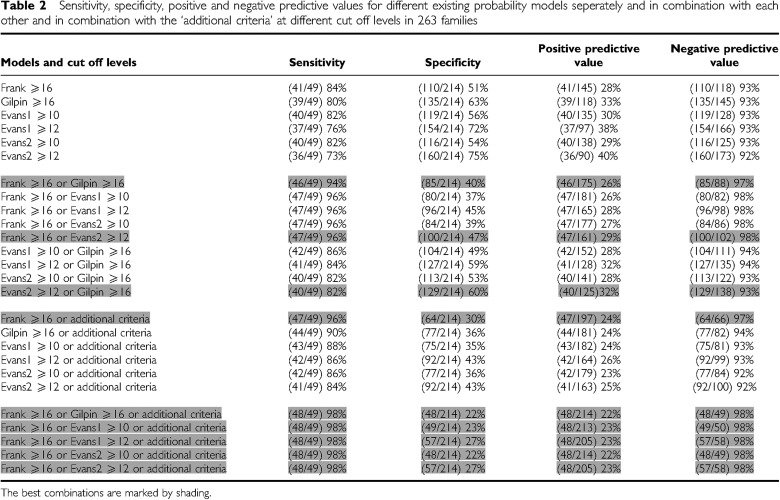
Sensitivity, specificity, positive and negative predictive values for different existing probability models seperately and in combination with each other and in combination with the ‘additional criteria’ at different cut off levels in 263 families

**Table 3:** Additional selection criteria specific for small BRCA mutation positive families

1.	Both breast and ovarian cancer (one or both diagnosed before age <60 year)
2.	Male with breast cancer (at any age)
3.	Bilateral breast cancer (diagnosed before age 45 years)
4.	Ovarian cancer diagnosed before age 40 years
5.	Breast cancer diagnosed before age 35 years
