# Supplementary material for: Optimal selection for BRCA1 and BRCA2 mutation testing using a combination of ‘easy to apply’ probability models
Source: Br J Cancer. 2006 Aug 15;95(6):757–62. doi: 10.1038/sj.bjc.6603306 (PMC2360521; doi:10.1038/sj.bjc.6603306)
Supplement: Supplementary data Table 1 [file 95-6603306x1.doc]

# Supplementary online material

**Table 1**  Sensitivity, specificity, positive and negative predictive value for the Frank, Gilpin, Evans1, Evans2 and additional selection criteria in 263 families.

| *Models and cut off levels* | *Sensitivity* | *Specificity* | *Positive predictive value* | *Negative predictive value* |
| --- | --- | --- | --- | --- |
| Frank ≥ 10 | (41/49) 84% | (87/214) 41% | (41/168) 24% | (87/95) 92% |
| Frank ≥ 16 | (41/49) 84% | (110/214) 51% | (41/145) 28% | (110/118) 93% |
| Frank ≥ 18 | (30/49) 61% | (174/214) 81% | (30/70) 43% | (174/193) 90% |
|  |  |  |  |  |
| Gilpin ≥ 10 | (47/49) 96% | (15/214) 7% | (47/246) 19% | (15/17) 88% |
| Gilpin ≥ 12 | (44/49) 90% | (52/214) 24% | (44/206) 21% | (52/57) 91% |
| Gilpin ≥ 14 | (41/49) 84% | (96/214) 45% | (41/159) 25% | (96/104) 92% |
| Gilpin ≥ 16 | (39/49) 80% | (135/214) 63% | (39/118) 33% | (135/145) 93% |
| Gilpin ≥ 18 | (33/49) 67% | (168/214) 79% | (33/79) 42% | (168/184) 91% |
|  |  |  |  |  |
| Evans1 ≥ 10 | (40/49) 82% | (119/214) 56% | (40/135) 30% | (119/128) 93% |
| Evans1 ≥ 12 | (37/49) 76% | (154/214) 72% | (37/97) 38% | (154/166) 93% |
| Evans2 ≥ 10 | (40/49) 82% | (116/214) 54% | (40/138) 29% | (116/125) 93% |
| Evans2 ≥ 12 | (36/49) 73% | (160/214) 75% | (36/90) 40% | (160/173) 92% |

As there was no difference between the parameters between the Frank ≥ 16, Frank ≥ 14 and Frank ≥ 12 cut off levels, the latter calculations are left out in this table

**Table 2**  Sensitivity, specificity, positive and negative predictive value for combinations of the different selection criteria and different cut off levels in 263 families.

| Models and cut off levels | *Sensitivity* | *Specificity* | *Positive predictive value* | *Negative predictive value* |
| --- | --- | --- | --- | --- |
|  |  |  |  |  |
| Frank ≥ 10 or Gilpin ≥ 12 | (48/49) 98% | (36/214) 17% | (48/226) 21% | (36/37) 97% |
| Frank ≥ 10 or Gilpin ≥ 16 | (46/49) 94% | (74/214) 35% | (46/186) 25% | (74/77) 96% |
| Frank ≥ 10 or Evans1 ≥ 10 | (47/49) 96% | (71/214) 33% | (47/190) 25% | (71/73) 97% |
| Frank ≥ 10 or Evans1 ≥ 12 | (47/49) 96% | (80/214) 37% | (47/181) 26% | (80/82) 98% |
| Frank ≥ 10 or Evans2 ≥ 10 | (47/49) 96% | (73/214)34% | (47/188) 25% | (73/75) 97% |
| Frank ≥ 10 or Evans2 ≥ 12 | (47/49) 96% | (83/214) 39% | (47/178) 26% | (83/85) 98% |
| Frank ≥ 16 or Gilpin ≥ 12 | (48/49) 98% | (40/214) 19% | (48/222) 22% | (40/41) 98% |
| Frank ≥ 16 or Gilpin ≥ 16 | (46/49) 94% | (85/214) 40% | (46/175) 26% | (85/88) 97% |
| Frank ≥ 16 or Evans1 ≥ 10 | (47/49) 96% | (80/214) 37% | (47/181) 26% | (80/82) 98% |
| Frank ≥ 16 or Evans1 ≥ 12 | (47/49) 96% | (96/214) 45% | (47/165) 28% | (96/98) 98% |
| Frank ≥ 16 or Evans2 ≥ 10 | (47/49) 96% | (84/214) 39% | (47/177) 27% | (84/86) 98% |
| Frank ≥ 16 or Evans2 ≥ 12 | (47/49) 96% | (100/214) 47% | (47/161) 29% | (100/102) 98% |
| Evans1 ≥ 10 or Gilpin ≥ 12 | (45/49) 92% | (47/214) 22% | (45/212) 21% | (47/51) 92% |
| Evans1 ≥ 12 or Gilpin ≥ 12 | (44/49) 90% | (50/214) 23% | (44/208) 21% | (50/55) 91% |
| Evans2 ≥ 10 or Gilpin ≥ 12 | (44/49) 90% | (49/214) 23% | (44/209) 21% | (49/54) 91% |
| Evans2 ≥ 10 or Gilpin ≥ 12 | (44/49) 90% | (51/214) 24% | (44/207) 21% | (51/56) 91% |
| Evans1 ≥ 10 or Gilpin ≥ 16 | (42/ 49) 86% | (104/214) 49% | (42/152) 28% | (104/111) 94% |
| Evans1 ≥ 12 or Gilpin ≥ 16 | (41/49) 84% | (127/214) 59% | (41/128) 32% | (127/135) 94% |
| Evans2 ≥ 10 or Gilpin ≥ 16 | (40/49) 82% | (113/214) 53% | (40/141) 28% | (113/122) 93% |
| Evans2 ≥ 12 or Gilpin ≥ 16 | (40/49) 82% | (129/214) 60% | (40/125)32% | (129/138) 93% |
| Frank ≥ 16 or Gilpin ≥ 16 or Evans1 ≥ 10 | (47/49) 96% | (73/214) 34% | (47/188) 25% | (73/75) 97% |
| Frank ≥ 16 or Gilpin ≥ 16 or Evans1 ≥ 12 | (47/49) 96% | (82/214)38% | (47/179) 26% | (82/84) 98% |
| Frank ≥ 16 or Gilpin ≥ 16 or Evans2 ≥ 10 | (47/49) 96% | (76/214) 36% | (47/185) 25% | (76/78) 97% |
| Frank ≥ 16 or Gilpin ≥ 16 or Evans2 ≥ 12 | (47/49) 96% | (84/214) 39% | (47/177) 27% | (84/86) 98% |
| Frank ≥ 18 or Gilpin ≥ 16 or Evans1 ≥ 10 | (43/49) 88% | (101/214) 47% | (43/156) 28% | (101/107) 94% |
| Frank ≥ 18 or Gilpin ≥ 16 or Evans1 ≥ 12 | (42/49) 86% | (119/214) 56% | (42/137) 31% | (119/126) 94% |
| Frank ≥ 18 or Gilpin ≥ 16 or Evans2 ≥ 10 | (42/49) 86% | (110/214) 51% | (42/146) 29% | (110/117) 94% |
| Frank ≥ 18 or Gilpin ≥ 16 or Evans2 ≥ 12 | (42/49) 86% | (122/214) 57% | (42/134) 31% | (122/129) 95% |
| Frank ≥ 16 or Gilpin ≥ 18 or Evans1 ≥ 10 | (47/49) 96% | (79/214) 37% | (47/182) 26% | (79/81) 98% |
| Frank ≥ 16 or Gilpin ≥ 18 or Evans1 ≥ 12 | (47/49) 96% | (93/214)43% | (47/168)28% | (93/95) 98% |
| Frank ≥ 16 or Gilpin ≥ 18 or Evans2 ≥ 10 | (47/49) 96% | (97/214) 45% | (47/164) 29% | (97/99) 98% |
| Frank ≥ 16 or Gilpin ≥ 18 or Evans2 ≥ 12 | (47/49) 96% | (83/214) 39% | (47/178) 26% | (83/85) 98% |
| Frank ≥ 18 or Gilpin ≥ 18 or Evans1 ≥ 10 | (41/49) 84% | (120/214) 56% | (41/135) 30% | (120/128) 94% |
| Frank ≥ 18 or Gilpin ≥ 18 or Evans1 ≥ 12 | (41/49) 84% | (136/214) 64% | (41/119) 34% | (136/144) 94% |
| Frank ≥ 18 or Gilpin ≥ 18 or Evans2 ≥ 10 | (41/49) 84% | (120/214) 56% | (41/135) 30% | (120/128) 94% |
| Frank ≥ 18 or Gilpin ≥ 18 or Evans2 ≥ 12 | (41/49) 84% | (141/214) 66% | (41/114) 36% | (141/149) 95% |

**Table 3**  Sensitivity, specificity, positive and negative predictive values for the “additional criteria” in combination with the Gilpin, Frank, Evans1 and Evans2 criteria at different cut off levels in 263 families.

| *Models and cut off levels* | *Sensitivity* | *Specificity* | *Positive predictive value* | *Negative predictive value* |
| --- | --- | --- | --- | --- |
|  |  |  |  |  |
| Additional criteria | (10/17) 59% | (108/174) 62% | (10/76) 13% | (108/115) 94% |
|  |  |  |  |  |
| Frank ≥ 10 or additional criteria | (47/49) 96% | (52/214) 24% | (47/209) 22% | (52/54) 96% |
| Frank ≥ 12 or additional criteria | (47/49) 96% | (64/214)30% | (47/197) 24% | (64/66) 97% |
| Frank ≥ 16 or additional criteria | (47/49) 96% | (64/214) 30% | (47/197) 24% | (64/66) 97% |
| Frank ≥ 18 or additional criteria | (42/49) 86% | (98/214) 46% | (42/158) 27% | (98/105) 93% |
|  |  |  |  |  |
| Gilpin ≥ 14 or additional criteria | (46/49) 94% | (55/214) 26% | (46/205) 22% | (55/58) 95% |
| Gilpin ≥ 16 or additional criteria | (44/49) 90% | (77/214) 36% | (44/181) 24% | (77/82) 94% |
| Gilpin ≥ 18 or additional criteria | (40/49) 82% | (95/214) 44% | (40/159) 25% | (95/104) 91% |
|  |  |  |  |  |
| Evans1 ≥ 10 or additional criteria | (43/49) 88% | (75/214) 35% | (43/182) 24% | (75/81) 93% |
| Evans1 ≥ 12 or additional criteria | (42/49) 86% | (92/214) 43% | (42/164) 26% | (92/99) 93% |
| Evans2 ≥ 10 or additional criteria | (42/49) 86% | (77/214) 36% | (42/179) 23% | (77/84) 92% |
| Evans2 ≥ 12 or additional criteria | (41/49) 84% | (92/214) 43% | (41/163) 25% | (92/100) 92% |
|  |  |  |  |  |
| Frank ≥ 16 or Gilpin ≥ 16 or additional criteria | (48/49) 98% | (48/214) 22% | (48/214) 22% | (48/49) 98% |
| Frank ≥ 16 or Gilpin ≥ 18 or additional criteria | (47/49) 96% | (59/214) 28% | (47/202) 23% | (59/61) 97% |
| Frank ≥ 18 or Gilpin ≥ 16 or additional criteria | (45/49) 92% | (73/214) 34% | (45/186) 24% | (73/77) 95% |
| Frank ≥ 18 or Gilpin ≥ 18 or additional criteria | (43/49) 88% | (87/214) 41% | (43/170) 25% | (87/93) 94% |
|  |  |  |  |  |
| Frank ≥ 16 or Evans1 ≥ 10 or additional criteria | (48/49) 98% | (49/214) 23% | (48/213) 23% | (49/50) 98% |
| Frank ≥ 16 or Evans1 ≥ 12 or additional criteria | (48/49) 98% | (57/214) 27% | (48/205) 23% | (57/58) 98% |
| Frank ≥ 18 or Evans1 ≥ 10 or additional criteria | (43/49) 88% | (72/214) 34% | (43/185) 23% | (72/78) 92% |
| Frank ≥ 18 or Evans1 ≥ 12 or additional criteria | (43/49) 88% | (85/214) 40% | (43/172) 25% | (85/91) 93% |
|  |  |  |  |  |
| Frank ≥ 16 or Evans2 ≥ 10 or additional criteria | (48/49) 98% | (48/214) 22% | (48/214) 22% | (48/49) 98% |
| Frank ≥ 16 or Evans2 ≥ 12 or additional criteria | (48/49) 98% | (57/214) 27% | (48/205) 23% | 57/58) 98% |
| Frank ≥ 18 or Evans2 ≥ 10 or additional criteria | (43/49) 88% | (74/214) 35% | (43/183) 23% | (74/80) 93% |
| Frank ≥ 18 or Evans2 ≥ 12 or additional criteria | (43/49) 88% | (85/214) 40% | (43/172) 25% | (85/91) 93% |
|  |  |  |  |  |
| Gilpin ≥ 16 or Evans1 ≥ 10 or additional criteria | (45/49) 92% | (63/214) 29% | (45/196) 23% | (63/67) 94% |
| Gilpin ≥ 16 or Evans1 ≥ 12 or additional criteria | (45/49) 92% | (74/214) 35% | (45/185) 24% | (74/78) 93% |
| Gilpin ≥ 18 or Evans1 ≥ 10 or additional criteria | (44/49) 90% | (71/214) 33% | (44/187) 24% | (71/76) 93% |
| Gilpin ≥ 18 or Evans1 ≥ 12 or additional criteria | (44/49) 90% | (86/214) 40% | (44/172) 26% | (86/91) 95% |
|  |  |  |  |  |
| Gilpin ≥ 16 or Evans2 ≥ 10 or additional criteria | (44/49) 90% | (66/214) 31% | (44/192) 23% | (66/71) 93% |
| Gilpin ≥ 16 or Evans2 ≥ 12 or additional criteria | (44/49) 90% | (74/214) 35% | (44/184) 24% | (74/79) 94% |
| Gilpin ≥ 18 or Evans2 ≥ 10 or additional criteria | (43/49) 88% | (73/214) 34% | (43/184) 23% | (73/79) 92% |
| Gilpin ≥ 18 or Evans2 ≥ 12 or additional criteria | (43/49) 88% | (86/214) 40% | (43/171) 25% | (86/92) 93% |
